# Retinal vein occlusion and the risk of acute myocardial infarction development: a 12-year nationwide cohort study

**DOI:** 10.1038/srep22351

**Published:** 2016-02-29

**Authors:** Tyler Hyungtaek Rim, John Seungsoo Han, Jaewon Oh, Dong Wook Kim, Seok-Min Kang, Eun Jee Chung

**Affiliations:** 1 Department of Ophthalmology, National Health Insurance Service Ilsan Hospital, Goyang, Gyeonggi, Korea; 2 Department of Ophthalmology, Severance Hospital, Institute of Vision Research, Yonsei University College of Medicine, Seoul, Korea; 3 Division of Cardiology, Severance Cardiovascular Hospital, Cardiovascular Research Institute, Yonsei University College of Medicine, Seoul, Korea; 4 Department of Policy Research Affairs, National Health Insurance Service Ilsan Hospital, Goyang, Gyeonggi, Korea

## Abstract

The goal of this study was to evaluate the risk of developing acute myocardial infarction (AMI) following retinal vein occlusion (RVO). A retrospective cohort study was performed from the National Health Insurance Service and comprised 1,025,340 random subjects who were followed from 2002 to 2013. Patients with RVO in 2002 were excluded. The RVO group was composed of patients who received an initial RVO diagnosis between January 2003 and December 2007 (n = 1677). The comparison group was selected (five patients per RVO patient; n = 8367) using propensity score matching according to sociodemographic factors and the year of enrolment. Each patient was tracked until 2013. The Cox proportional hazard regression model was used. AMI developed in 7.6% of the RVO group and 5.3% of the comparison group (p < 0.001) for 7.7 median follow-up periods. RVO increased the risk of AMI development [hazard ratio (HR) = 1.25; 95% Confidence Interval (CI) 1.02 to 1.52]. In the subgroup analysis, RVO patients aged <65 years and the males within this age group had an adjusted HR of 1.47 (95% CI 1.10 to 1.98) and an adjusted HR of 2.00 (95% CI 1.38 to 2.91) for AMI development, respectively. RVO was significantly associated with AMI development.

Retinal vein occlusion (RVO) is classified into central RVO (CRVO) and branch RVO (BRVO)^1^ and is an ischemic event that affects the retina, which is a structure derived from the central nervous system^2^. RVO is associated with thrombosis in the vein due to compression by an adjacent atherosclerotic artery, an increased homocysteine level, and rheological alterations, including decreased blood flow and increased blood viscosity^3^,^4^,^5^,^6^. RVO is commonly associated with conditions such as hypertension, diabetes mellitus, and a wide variety of haematological disorders^7^,^8^. These risk factors for RVO are also risk factors for fatal systemic vascular diseases, such as cardiovascular or cerebrovascular disease^9^,^10^. Several studies have analysed the association between RVO and cardiovascular disease, but the results have been inconsistent^11^,^12^,^13^,^14^,^15^,^16^,^17^. However, recent well-designed, large-scale studies in Taiwan^14^ and the United States^15^ did not show a significant association between RVO and the prospective risk of acute myocardial infarction (AMI) development.

In this study, we investigated the association between RVO and the prospective risk of AMI development using a nationwide representative sample of 1,025,340 adults from the National Health Insurance Service National Sample Cohort 2002–2013 (NHIS-NSC 2002–2013) in South Korea.

## Methods

### Ethical approval

This study adhered to the tenets of the Declaration of Helsinki, and the NHIS-NCS 2002–2013 project was approved by the Institutional Review Board of the Korean National Health Insurance Service. This study design was reviewed and approved by the Institutional Review Board of the National Health Insurance Service, Ilsan Hospital, Gyeonggi-do, Korea. Written informed consent was waived.

### Data Sources

Korea has maintained a nationwide health insurance system since 1963 under the Korean National Health Insurance Service (KNHIS), and nearly all of the data in the health system are centralized in large databases. Claims are accompanied by data regarding diagnostic codes, procedures, prescription drugs, personal information, and medical costs. No patient health care records are duplicated or omitted because all Korean residents receive a unique identification number at birth. Furthermore, the KNHIS uses the Korean Classification of Diseases (KCD), which is a system similar to the International Classification of Diseases (ICD). This study used the NHIS-NSC 2002–2013, which comprised 1,025,340 nationally representative random subjects accounting for approximately 2.2% of the entire population in 2002.

### Study Population

The RVO group included all patients who received inpatient and outpatient care between January 2003 and December 2007 for an initial diagnosis of RVO [KCD code H34.8 corresponding to ICD-9-clinical modification (CM) code 362.35, CRVO, or 362.36, venous tributary (branch) occlusion]. We excluded subjects treated for RVO in ambulatory and inpatient care in 2002 to eliminate patients with a chronic condition and to ensure that the RVO group included only subjects with new episodes. For the same reason, patients who received a diagnosis of AMI in 2002 (KCD code I21 corresponding to ICD-9-CM code 410, AMI) were also considered to have a chronic condition and were excluded. We included patients who were diagnosed with RVO prior to their AMI based on the patient’s first visit. Finally, 1677 eligible patients with RVO in 2003–2007 were identified after excluding potential pre-existing cases of RVO and/or AMI. These patients were regarded as new incident RVO. We selected 8367 patients (five per RVO patient) from the database; these patients were matched to the RVO group using propensity score matching. Each patient was tracked on the basis of his or her ambulatory visit date and the first visit date for inpatient care over the 11 year period from 2003 to 2013 to identify patients who developed AMI.

### Comorbidities

We defined hypertension (KCD code I10 corresponding to ICD-9-CM code 401, essential hypertension), diabetes mellitus (KCD code E10–E14 corresponding to ICD–9–CM code 250, Diabetes mellitus), chronic renal failure (KCD code N18 corresponding to ICD-9-CM code 585, Chronic kidney disease), hyperlipidaemia (KCD code E78.0–78.5 corresponding to ICD–9–CM code 272.0, pure hypercholesterolemia, 272.1, pure hyperglyceridaemia, 272.2, mixed hyperlipidaemia, 272.3, hyperchylomicronaemia, and 272.4, other and unspecified hyperlipidaemia), and stroke (KCD code I60–I63 corresponding to ICD-9-CM code 430–434, cerebrovascular disease) as comorbidities because they were all known risk factors for AMI^10^,^18^. The comorbidities were defined as a diagnosis of any of these conditions between 2003 and 2013 and prior to the AMI diagnosis.

### Statistical Analysis

Descriptive statistics of the study population are presented. Propensity score matching was performed; to predict RVO occurrence, propensity scores were estimated using logistic regression to control for the sociodemographic factors age (<50, 50–59, 60–69, 70–79, and ≥80 years), gender, residential area (the Korean metropolitan area Seoul, the 2nd area including the largest province, the 3rd area including the second largest city and the 2nd and 3rd largest provinces, and the 4th area including other areas), and household income (≤30%, 30–70%, and 70–100% of the median). Matching was performed using the Greedy 8 → 1 digit matching macro with the estimated propensity score in each year from 2003 to 2007^19^. To identify the hazards associated with AMI, hazard ratios (HRs) and 95% confidence intervals (CIs) were calculated via univariable and multivariable Cox proportional hazard regression analyses. The overall AMI-free survival rate was determined using the Kaplan-Meier curve for the 11-year follow-up period. Follow-up began at the first date of RVO diagnosis or at a randomly selected index date in the matched year for the comparison group. Follow-up ended at the last visit date up to 2013 for subjects without subsequent AMI or the first date of AMI diagnosis. A significance level of p < 0.05 was selected. The statistical packages SAS System for Windows, version 9.4 (SAS Institute Inc., Cary, NC, USA) and Stata/MP version 14.0 (StataCorp, College Station, TX, USA) were used to perform the analyses in this study.

## Results

Table 1 displays the characteristics of the study population for the two cohorts (the RVO group and the comparison group). The subjects with RVO were more likely to experience AMI (p < 0.001), hypertension (p < 0.001), diabetes mellitus (p < 0.001), chronic renal failure (p < 0.001), hyperlipidaemia (p < 0.001) and stroke (p = 0.020) than the comparison group. No significant difference in the year of RVO diagnosis, age, gender, residential area, or household income was detected between the groups because these variables were used for sample matching.

Table 2 displays the HRs for AMI during the up to 11-year follow-up period calculated using univariable and multivariable Cox regression models. After adjusting for age, household income, and comorbidities including hypertension, diabetes mellitus, chronic renal failure, hyperlipidaemia, and stroke, RVO was associated with the prospective development of AMI (HR = 1.25, 95% CI 1.02 to 1.52) based on the multivariable Cox regression analysis. Hypertension (HR = 2.64, 95% CI 1.98 to 3.50), chronic renal failure (HR = 1.83, 95% CI 1.35 to 2.49), and stroke (HR = 1.59, 95% CI 1.02 to 2.46) were associated with the development of AMI. In terms of sociodemographic characteristics, increasing age was significantly associated with the development of AMI and the female gender was associated with a lower incidence of AMI.

Table 3 shows the subgroup analysis of age stratified into two subgroups (<65 and ≥65 years) based on the multivariable Cox regression analysis. RVO patients aged <65 years had a significantly higher risk for the development of AMI (HR = 1.47, 95% CI 1.10 to 1.98). However, RVO was no longer a significant predictor for AMI development in the ≥65 year age group. In both age groups (<65 years and ≥65 years), hypertension and chronic renal failure were associated with AMI. A gender difference existed in both age groups.

Table 4 shows the gender subgroup analysis in the <65 year age group. Male RVO patients <65 years had a higher risk for the development of AMI (HR = 2.00, 95% CI 1.38 to 2.91). Conversely, RVO was no longer a significant predictor for the development of AMI in females aged <65 years. Hypertension was associated with an increased risk of AMI for both genders. Chronic renal failure was associated with an increased risk of AMI in males, and stroke was associated with an increased risk of AMI in females.

**Figure 1:**
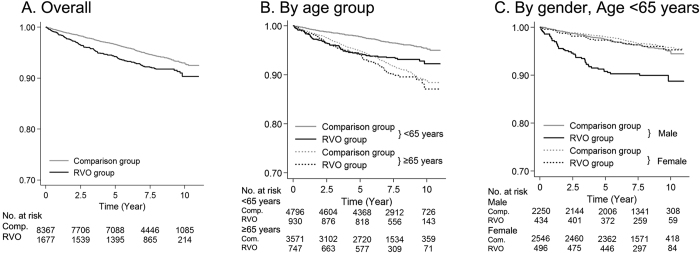
Acute myocardial infarction (AMI)–free survival rate for the retinal vein occlusion (RVO) group and the comparison group (Comp.) over the 11-year follow-up period. The overall AMI-free rate (**A**) AMI-free rate by age group (<65 years and ≥65 years) (**B**) and AMI-free rate by gender in subjects aged <65 years (**C**) are shown.

Figure 1 displays the Kaplan-Meier curve for the AMI-free survival rate. The median follow-up was 7.7 years. A total of 73,411 person-years were examined (12,188 person-years for the RVO group and 61,223 person-years for the matched controls). AMI occurred at a rate of 10.5 per 1000 person-years in the RVO group and 7.3 per 1000 person-years in the comparison group (for a total of 7.8 per 1000 person-years). The log rank tests indicated that patients with RVO developed AMI significantly more frequently than the comparison group overall (Fig. 1A; p < 0.001), in the age group <65 years (Fig. 1B; p < 0.001), and in males aged <65 years (Fig. 1C; p < 0.001). Figure 1B indicated that RVO was an important predictor for AMI in adults younger than 65 years of age because the difference in the AMI-free survival rate between the RVO group and the comparison group in adults older than 65 years was much smaller than that observed in younger adults. Figure 1C indicated that RVO was an important predictor for AMI in males aged <65 years, whereas the AMI-free survival rate was similar between females with RVO and females without RVO.

## Discussion

In this study, we examined the association between RVO and AMI in 10,044 sociodemographically matched subjects extracted from a nationwide 12-year longitudinal cohort database of 1,025,340 Koreans. We found that RVO patients exhibited a higher risk for the development of AMI during an up to 11-year follow-up period after adjusting for comorbidities and sociodemographic factors.

Despite remarkable progress in medical science, myocardial infarction remains an important health problem^20^, and the incidence of AMI has been on the rise in Asia (which is opposite to the trend reported in the United States) due to the Westernization of the diet and the aging of the population^21^,^22^,^23^. The clinical importance of cardiovascular disabilities and the possibility of the association between RVO and AMI development or myocardial infarction mortality have spurred several previous studies, but the results have been inconsistent^11^,^12^,^13^,^14^,^15^,^16^,^17^,^24^,^25^,^26^,^27^. A relatively small sample size, short follow-up period, and insufficiently representative samples generated from a single centre constitute limitations that may have caused the inconsistent results in previous studies. In terms of myocardial infarction mortality, a pooled data analysis of two Caucasian population-based cohorts reported that baseline RVO was associated with a higher cardiovascular mortality rate in persons aged <70 years after adjusting for age, gender, body mass index, hypertension, diabetes, smoking, glaucoma, and study site^26^. However, there was no significant difference in the mortality risk between persons of all ages with and without RVO. Another study using the Framingham algorithm estimated higher mortality resulting from cardiovascular disease among patients with RVO^13^. In contrast, another study found that mortality was not higher in CRVO patients^12^. However, cardiovascular or overall mortality may not fully explain the association between RVO and AMI, and the mortality rate is not equal to AMI development when interpreting the results of these studies.

Recently, well–designed population-based studies were conducted to evaluate the association between AMI and RVO in several regions. A methodologically similar study using a Taiwanese nationwide population base found that RVO patients did not have a significantly higher rate of AMI during a 3-year follow-up period after adjusting for confounding factors (adjusted HR = 1.17 for BRVO group and adjusted HR = 2.33 for CRVO group compared to the control)^14^. When the results of that study were compared with those of the current study, several differences were noted. In the Taiwan study, the total number of eligible RVO patients over the four year period was 591, of which 11 patients suffered AMI (1.86% of the RVO patients). This number was much smaller than the 1,677 RVO patients eligible over the five year period and the 128 patients who suffered AMI (7.6% of the RVO patients examined) in the current study. According to previous studies based on the national health insurance database, the incidence and rates of AMI in Korea (118.4 and 91.8 per 100,000 persons, respectively, in 2007) were higher than those in Taiwan (62.4 and 55.7 per 100,000 persons, respectively, in 2008)^21^,^22^. The small number of cases and relatively short study period in Taiwan may have resulted in insufficient power to detect the associations.

Another large study comparing 4500 patients with RVO and 13,500 controls based on a health care claims database in the US reported that the event rates for AMI were similar in patients with RVO and the controls after adjusting for confounding factors (adjusted rate ratio = 1.03, 95% CI 0.75 to 1.42)^15^. In the subgroup analysis, males and patients younger than 65 years with RVO had adjusted HRs of 1.6 and 1.9 for AMI, respectively, compared with the controls. The present study showed similar results in terms of a stronger association between RVO and AMI in younger adults and/or males. RVO was associated with an increased risk of AMI (HR = 1.25, 95% CI 1.02 to 1.52), and our subgroup analysis showed that RVO patients aged <65 years and males aged <65 years had a significantly higher risk (HR = 1.47, 95% CI 1.10 to 1.98; and HR = 2.00, 95% CI 1.38 to 2.91, respectively) for the development of AMI after adjusting for possible confounding factors. Finally, the present study showed a clearer association between RVO and AMI than other studies because our study had a longer duration with an up to 11-year follow-up period (median 7.7 years), a large number of RVO patients (70.4% power with a sample size of 10,000), and a relatively increasing trend in the AMI incidence in Korea (7.8 occurrence per 1,000 person-year) that was in contrast to the decreasing trend found in other developed Western countries^21^,^22^,^23^.

A previous INTERHEART global case-control study from 52 countries showed that traditional risk factors were generally stronger in association with AMI development among younger adults compared to older adults and that hypertension was more strongly associated with AMI in females than males^28^. Our results were consistent with the differences in age- and gender-based associations between comorbidities and AMI. In younger adults aged <65 years, hypertension (HR = 3.36) was the most important risk factor for AMI and RVO (HR = 1.47), chronic renal failure (HR = 2.00) and stroke (HR = 1.77) increased the risk of AMI. In older adults aged ≥65 years, RVO was no longer a significant predictor for AMI, whereas hypertension (HR = 2.35) and chronic renal failure (HR = 1.77) were associated with AMI. Moreover, in younger adults aged <65 years, RVO was associated with the male gender (HR = 2.00) and not the female gender (HR = 0.93, Table 4). Figures 1B,C described the importance of age and gender in using RVO as a predictor for AMI. Figure 1C showed that RVO was a particularly important risk factor for AMI in men aged <65 years in addition to other classic systemic comorbidities. The pathophysiology responsible for RVO development may differ according to age. It is generally recognized that retinal vein inflammation is a primary cause of RVO in young patients^29^. In older patients, RVO is more related to compression of the retinal vein due to atherosclerotic changes in the neighbouring arterioles in which a retinal vein is compressed by an adjacent retinal artery, resulting in thrombus formation and retinal ischemia^8^,^30^. These age-dependent RVO mechanisms may be associated with differences in the AMI rate in different age groups. RVO could be another candidate in the risk profiles for the development of AMI in young males. Although overall morbidity and mortality resulting from AMI have decreased, AMI hospitalization rates for young patients have not declined over the past decade in the United States^31^. In Korea, AMI rates in the young have increased, whereas the AMI rates in the older population have decreased^32^. Considering these medical backgrounds, we should pay more attention to young patients with RVO as a high risk cardiovascular disease group, especially for the risk of an AMI. AMI can occur during the natural course of coronary atherosclerosis, and coronary artery occlusion from plaques vulnerable to rupture or erosion is the most common cause of myocardial infarction^33^,^34^. In our recently published report, we showed that retinal vessel occlusion increased the risk of stroke (HR = 1.48 by RVO; HR = 1.78 by retinal artery occlusion)^35^,^36^. In this study, RVO increased the risk of AMI. These results may be explained by the similarity in the occlusive mechanism of both stroke and AMI. Improved blood pressure control might also be helpful in decreasing the risk of AMI in RVO patients. However, this epidemiological study does not provide evidence for the pathophysiology behind this finding.

### Strengths and limitations of the study

We examined the association between AMI and RVO in a nationwide, standardized 12-year longitudinal cohort based on a large sample size of 10,044 patients and controls. A relatively large number of RVO patients and a long study period are the main strengths of the present study.

The limitations of this study include: 1) the possibility of misclassification of diagnoses for RVO, AMI, or comorbidities, 2) possible underreporting of asymptomatic RVO or AMI patients who did not receive medical care, 3) the possibility of delayed visits to the ophthalmologist or cardiologist and thus delayed diagnosis of RVO or AMI, respectively, 4) the possibility that chronic RVO patients are not fully excluded, 5) the inability to collect other important health-related information, such as alcohol consumption, 6) the possibility that a medical claim may have included biased controls compared to general population-based controls who neither received medical care nor had a specific diagnosis, 7) the possibility that racial differences may exist in the South Korean population and 8) the lack of access to data regarding the types of RVO (central or branch) or classification of AMI (non-ST elevation MI, or ST elevation MI).

The most important limitation of this study was that the RVO diagnoses were defined based on KCD codes, which might be inaccurate compared with the diagnoses obtained from a medical chart that included imaging results and thus might make misdiagnoses possible. The validity of the medical insurance claims data regarding RVO in Korea were discussed in our previous RVO study based on the NHIS-NSC 2002–2010 database^35^. A previous study revealed that the accuracy for diagnosing AMI using the ICD-10 codes in the Korean medical insurance claims data was 71.4% according to the World Health Organization and 73.1% according to the American College of Cardiology/the European Society of Cardiology Committee criteria; moreover, the reliability was fair to good^37^. To verify the NHIS-NSC 2002–2013 data, the prevalence of 20 major diseases for each year was calculated to compare the trends in the annual changes in prevalence. KNHIS confirmed that the prevalence of each disease was similar, thereby validating the quality of the data. Additionally, the NHIS-NSC 2002–2013 primarily included medical claims but only partially included other health examination data, including the body mass index and behavioural risk factors such as the smoking status. In this respect, these possible confounding factors could not be controlled. Only limited information is available in the NHIS-NSC 2002–2013 claims database, such as the procedure code or diagnosis code. Unfortunately, it was impossible to distinguish between branch and central RVO, although the two conditions were known to have different risk factors^38^. Our explanatory analyses based on RVO patients who received photocoagulation laser therapy (RVO patients with laser) and who had not ever received photocoagulation laser therapy (RVO patients without laser) showed that the effect size (~HR) was greater in the RVO patients with laser therapy than in the RVO patients without laser therapy compared to the comparison group as a reference group (unadjusted HR = 1.60 for RVO patients with laser therapy and 1.37 for RVO patients without laser therapy). Therefore, further clinical studies are needed, including a stratified analysis of RVO types.

## Conclusions

RVO was associated with AMI development after adjusting for potential confounding conditions. The physician should keep in mind that RVO is an important predictor for AMI, especially in adult males aged <65 years. Ophthalmologists should be watchful for patients with RVO to control for known risk factors, such as hypertension and diabetes mellitus, and have the patient checked for these disorders if he or she is not already under the care of a primary care physician.

## Additional Information

**How to cite this article**: Rim, T. H. *et al*. Retinal vein occlusion and the risk of acute myocardial infarction development: a 12-year nationwide cohort study. *Sci. Rep.* **6**, 22351; doi: 10.1038/srep22351 (2016).

## Figures and Tables

**Table 1:** Characteristics of the study population comparison group (n = 8,367) and the retinal vein occlusion (RVO) group (n = 1,677).

Variables	Comparison group (column %)		RVO group (column %)		P value
Acute myocardial infarction					<0.001
No event	7,923	(94.7)	1549	(92.4)	
Event	444	(5.3)	128	(7.6)	
Hypertension					<0.001
No	2,796	(33.4)	282	(16.8)	
Yes	5,571	(66.6)	1395	(83.2)	
Diabetes mellitus					<0.001
No	3,984	(47.6)	584	(34.8)	
Yes	4,383	(52.4)	1093	(65.2)	
Chronic renal failure					<0.001
No	8,113	(97.0)	1578	(94.1)	
Yes	254	(3.0)	99	(5.9)	
Dyslipidaemia					<0.001
No	3,205	(38.3)	443	(26.4)	
Yes	5,162	(61.7)	1234	(73.6)	
Stroke					0.020
No	8,219	(98.2)	1633	(97.4)	
Yes	148	(1.8)	44	(2.6)	
**Variables for matching**					
Year					
2003	1,641	(19.6)	329	(19.6)	>0.999
2004	1,893	(22.6)	379	(22.6)	
2005	1,581	(18.9)	317	(18.9)	
2006	1,473	(17.6)	295	(17.6)	
2007	1,779	(21.3)	357	(21.3)	
Age group (year)					>0.999
<50	1,205	(14.4)	241	(14.4)	
50–59	1,963	(23.5)	393	(23.4)	
60–69	3,076	(36.8)	616	(36.7)	
70–79	1,662	(19.9)	334	(19.9)	
≥80	461	(5.5)	93	(5.6)	
Sex					0.987
Male	3,659	(43.7)	733	(43.7)	
Female	4,708	(56.3)	944	(56.3)	
Residence					>0.999
Seoul (metropolitan)	1,774	(21.2)	355	(21.2)	
2nd area	1,507	(18.0)	302	(18.0)	
3rd area	1,839	(22.0)	369	(22.0)	
4th area	3,247	(38.8)	651	(38.8)	
Household income					>0.999
0–30%	1,896	(22.7)	380	(22.7)	
30–70%	2,748	(32.8)	551	(32.9)	
70–100%	3,723	(44.5)	746	(44.5)	

Seoul, a metropolitan area in Korea; the 2nd area included the largest province; the 3rd area included the second largest city and two 2nd and 3rd largest provinces; and the 4th area included other areas.

**Table 2:** Univariable and multivariable Cox regression analysis for the overall incidence rate of acute myocardial infarction (n = 10,044).

Variables	Univariable Cox					Multivariable Cox				
	HR	(95% CI)			p–value	HR	(95% CI)			p–value
Group										
Comparison group	1(ref)					1(ref)				
RVO group	1.45	1.19	–	1.76	<0.001	1.25	1.02	–	1.52	0.029
Hypertension										
No	1(ref)					1(ref)				
Yes	3.71	2.84	–	4.85	<0.001	2.64	1.98	–	3.50	<0.001
Diabetes mellitus										
No	1(ref)					1(ref)				
Yes	1.50	1.26	–	1.78	<0.001	1.01	0.83	–	1.21	0.957
Chronic renal failure										
No	1(ref)					1(ref)				
Yes	2.62	1.94	–	3.53	<0.001	1.83	1.35	–	2.49	<0.001
Dyslipidaemia										
No	1(ref)					1(ref)				
Yes	1.44	1.19	–	1.74	<0.001	1.09	0.89	–	1.34	0.386
Stroke										
No	1(ref)					1(ref)				
Yes	2.13	1.38	–	3.29	0.001	1.59	1.02	–	2.46	0.039
Age group (year)										
<50	1(ref)					1(ref)				
50–59	1.91	1.27	–	2.89	0.002	1.61	1.06	–	2.43	0.026
60–69	3.43	2.35	–	5.02	<0.001	2.61	1.77	–	3.85	<0.001
70–79	4.41	2.98	–	6.52	<0.001	3.19	2.13	–	4.78	<0.001
≥80	6.44	4.04	–	10.25	<0.001	4.67	2.91	–	7.50	<0.001
Sex										
Male	1(ref)					1(ref)				
Female	0.81	0.69	–	0.95	0.012	0.73	0.62	–	0.86	<0.001
Residence										
Seoul (metropolitan)	1(ref)					1(ref)				
2nd area	1.02	0.78	–	1.33	0.879	1.08	0.83	–	1.41	0.564
3rd area	1.05	0.82	–	1.35	0.680	1.14	0.89	–	1.47	0.289
4th area	1.03	0.83	–	1.29	0.773	1.08	0.87	–	1.35	0.488
Household income										
0–30%	1(ref)					1(ref)				
30–70%	0.97	0.78	–	1.22	0.802	1.03	0.82	–	1.29	0.811
70–100%	1.00	0.81	–	1.23	0.973	0.93	0.75	–	1.15	0.502

CI = confidence interval; HR = hazard ratio; RVO = retinal vein occlusion.

Seoul, a metropolitan area in Korea; the 2nd area included the largest province; the 3rd area included the second largest city, and two 2nd and 3rd largest provinces; and the 4th area included other areas.

**Table 3:** Multivariable Cox regression analysis for the overall incidence rate of acute myocardial infarction according to age group (n = 10,044).

Variables	Age group									
	Age < 65 years (n = 5,726)					Age ≥ 65 years (n = 4,318)				
	HR	(95% CI)			p–value	HR	(95% CI)			p–value
Group										
Comparison group	1(ref)					1(ref)				
RVO group	1.47	1.10	–	1.98	0.010	1.05	0.80	–	1.37	0.749
Hypertension										
No	1(ref)					1(ref)				
Yes	3.36	2.30	–	4.90	<0.001	2.34	1.53	–	3.59	<0.001
Diabetes mellitus										
No	1(ref)					1(ref)				
Yes	0.97	0.73	–	1.29	0.835	1.06	0.83	–	1.36	0.632
Chronic renal failure										
No	1(ref)					1(ref)				
Yes	2.00	1.20	–	3.32	0.008	1.75	1.20	–	2.57	0.004
Dyslipidaemia										
No	1(ref)					1(ref)				
Yes	0.98	0.72	–	1.34	0.916	1.20	0.92	–	1.56	0.187
Stroke										
No	1(ref)					1(ref)				
Yes	1.77	0.90	–	3.46	0.097	1.45	0.81	–	2.58	0.211
Sex										
Male	1(ref)					1(ref)				
Female	0.68	0.53	–	0.88	0.004	0.78	0.63	–	0.97	0.028
Residence										
Seoul (metropolitan)	1(ref)					1(ref)				
2nd area	0.82	0.52	–	1.29	0.395	1.22	0.87	–	1.69	0.246
3rd area	1.38	0.94	–	2.02	0.102	0.98	0.70	–	1.36	0.884
4th area	1.25	0.88	–	1.77	0.211	0.93	0.69	–	1.24	0.603
Household income										
0–30%	1(ref)					1(ref)				
30–70%	1.04	0.74	–	1.45	0.829	1.01	0.74	–	1.38	0.949
70–100%	0.85	0.61	–	1.20	0.360	1.00	0.76	–	1.31	0.973

CI, confidence interval; HR, hazard ratio; RVO = retinal vein occlusion.

Seoul, a metropolitan area in Korea; the 2nd area included the largest province; the 3rd area included the second largest city, and two 2nd and 3rd largest provinces; and the 4th area included other areas.

**Table 4:** Multivariable Cox regression analysis for overall incidence rate of acute myocardial infarction according to gender in younger adults aged <65 years (n = 5,726).

Variables	Age < 65 years									
	Male (n = 2,684)					Female (n = 3,042)				
	HR	(95% CI)			p–value	HR	(95% CI)			p–value
Group										
Comparison group	1(ref)					1(ref)				
RVO group	2.00	1.38	–	2.91	<0.001	0.92	0.56	–	1.52	0.746
Hypertension										
No	1(ref)					1(ref)				
Yes	3.41	2.03	–	5.72	<0.001	3.30	1.90	–	5.73	<0.001
Diabetes mellitus										
No	1(ref)					1(ref)				
Yes	0.99	0.68	–	1.45	0.963	0.92	0.60	–	1.41	0.704
Chronic renal failure										
No	1(ref)					1(ref)				
Yes	1.98	1.04	–	3.75	0.037	1.99	0.86	–	4.60	0.108
Dyslipidaemia										
No	1(ref)					1(ref)				
Yes	1.06	0.70	–	1.60	0.780	0.89	0.55	–	1.44	0.631
Stroke										
No	1(ref)					1(ref)				
Yes	0.88	0.28	–	2.80	0.831	3.61	1.57	–	8.31	0.003
Residence										
Seoul (metropolitan)	1(ref)					1(ref)				
2nd area	0.98	0.53	–	1.82	0.957	0.65	0.33	–	1.28	0.213
3rd area	1.59	0.91	–	2.76	0.102	1.16	0.68	–	1.99	0.590
4th area	1.49	0.91	–	2.44	0.110	0.97	0.59	–	1.62	0.919
Household income										
0–30%	1(ref)					1(ref)				
30–70%	0.94	0.60	–	1.48	0.799	1.19	0.73	–	1.95	0.487
70–100%	0.80	0.51	–	1.28	0.353	0.96	0.58	–	1.61	0.890

CI, confidence interval; HR, hazard ratio; RVO = retinal vein occlusion.

Seoul, a metropolitan area in Korea; the 2nd area included the largest province; the 3rd area included the second largest city, and two 2nd and 3rd largest provinces; and the 4th area included other areas.
